# Outcomes and predictive factors of vital pulp therapy in a large-scale retrospective cohort study over 10 years

**DOI:** 10.1038/s41598-024-52654-8

**Published:** 2024-01-24

**Authors:** Saeed Asgary, Leyla Roghanizadeh, Mohammad Jafar Eghbal, Alireza Akbarzadeh Baghban, Anita Aminoshariae, Ali Nosrat

**Affiliations:** 1 Iranian Centre for Endodontic Research, Research Institute of Dental Sciences, Shahid Beheshti University of Medical Sciences, Tehran, Iran; 2 Dental Research Center, Research Institute of Dental Sciences, Shahid Beheshti University of Medical Science, Tehran, Iran; 3 Proteomics Research Center, Department of Biostatistics, School of Allied Medical Sciences, Shahid Beheshti University of Medical Sciences, Tehran, Iran; 4 Department of Endodontics, CWRU School of Dental Medicine, Cleveland, OH USA; 5 Division of Endodontics, Department of Advanced Oral Sciences and Therapeutics, University of Maryland, School of Dentistry, Baltimore, MD USA; 6 Private Practice, Centreville Endodontics, Centreville, VA USA

**Keywords:** Medical research, Risk factors

## Abstract

This cohort study evaluated the long-term success/survival of vital pulp therapies (VPTs) after carious pulp exposure in adult teeth. Additionally, factors influencing long-term success were identified. Teeth treated during 2011–2022 in a private clinic were studied with clinical/radiographic follow-ups. Data included patient demographics, tooth specifics, and treatment details. Outcomes were classified as success/failure based on clinical/radiographic findings, with tooth functionality determining the survival rate. Encompassing 1149 patients and 1257 VPT-treated teeth, the average monitoring period was 42.2 months. Overall VPTs’ survival and success rates were 99.1% and 91.6%, respectively. Success rates for 768 direct pulp cappings, 217 miniature pulpotomies, and 272 full pulpotomies were 91.9%, 92.6%, and 90.1%, respectively (*P* > 0.05). Influencing factors included symptomatic irreversible pulpitis (SIP; HR 1.974, 95% CI 1.242–3.137; *P* = 0.004), radiographic signs of apical periodontitis (AP; HR 2.983, 95% CI 1.961–4.540; *P* < 0.001), restoration type (HR 2.263, 95%CI 1.423–3.600; *P* = 0.001), and restoration surfaces (HR 1.401, 95%CI 1.034–1.899; *P* = 0.030). This study concludes that VPT techniques consistently exhibit high long-term success/survival rates in treating carious pulp exposures. Critical predictors include initial clinical signs of SIP/AP, caries extent, and use of composite restorations.

## Introduction

Vital pulp therapies (VPTs) have been employed to maintain the vitality/function of dental pulp tissue after damage due to various causes like caries, trauma, or dental procedures. With the modern approaches toward minimally invasive endodontic procedures for managing deep caries and exposed pulp, interest in VPT has seen a resurgence^1^. Notably, VPT techniques such as indirect/direct pulp capping (IPC/DPC), and miniature/partial/full pulpotomy (MP/PP/FP) have yielded promising results, prompting the American Association of Endodontists to release a Position Statement in 2021 recommending VPT as a viable option for mature permanent teeth showing signs of irreversible pulpitis (IP).

A 2011 systematic review evaluating the efficacy of VPT observed that cariously exposed vital pulps can be successfully managed with DPC/PP/FP, boosting success rates ranging from 72.9 to 99.4%^2^. Another comprehensive analysis focused specifically on FP outcomes in mature permanent teeth displaying symptoms of IP reported a radiographic success rate of 88.39% over a 3-year follow-up^3^. Overall, the clinical and radiographic success of VPT with different techniques/materials has been reported to be 81–96% in several randomized controlled trials and systematic reviews^4–8^.

One study on 273 permanent teeth retrospectively investigated factors shaping FP success rates. While dentinal bridge formation positively influenced outcomes, the use of direct composite restorations negatively impacted results. Over time, an initial 89% success rate after the first year decreased to 63% by the tenth year^9^.

In the present landscape, both VPT and root canal therapy (RCT) stand as credible, viable, and successful interventions for treating permanent teeth irrespective of the presence of IP or apical periodontitis (AP; e.g., widened PDL)^10,11^. It has been conventionally believed that teeth with IP or AP could only be effectively treated by RCT; however, recent studies highlight challenges and variable success rates associated with different modern RCT approaches^12,13^.

Yet, VPT has distinct advantages, being more accessible, cost-effective, safer, simpler, minimally invasive, and requiring less time than RCTs^14^. Despite these benefits, a noticeable gap exists in the literature concerning long-term VPT outcome studies with large sample sizes. As highlighted by previous researchers, observational studies involving sizable patient samples, combined with multivariate statistical models, provide the accuracy needed to pinpoint factors influencing the desired outcome^15^.

Considering these insights and the recognized limitations in current literature, the primary objectives of our study for treating carious pulp exposures were twofold: (1) to discern long-term treatment outcomes, including survival and success rates of VPTs administered to a comprehensive cohort of patients, and (2) to shed light on prognostic factors (e.g., age, sex, tooth type, pulpal/periapical diagnoses, VPT type, restoration material, and restoration surfaces) dictating tooth survival and success post VPTs. In alignment with these objectives, the null hypothesis of the study was formulated as follows: The accompanying factors of VPTs encompassing the patient’s age and sex, tooth type, pre-treatment pulpal/periapical diagnoses, VPT type, type of restoration material, and restoration surfaces do not exert a significant influence on the outcomes of the VPTs specifically the treatment success and survival rates.

## Methods

### Study design

We conducted a retrospective cohort study of patients who underwent various VPTs due to carious pulp exposure in their mature permanent teeth after caries excavation. The study received approval from the Research Institute for Dental Sciences (RIDS) at Shahid Beheshti University of Medical Sciences. All methods were performed in accordance with ethical standards and research protocols. We used the recommendations of STROBE cohort reporting guidelines to report the data^16^.

### Setting

We screened patients from Mehr Dental Clinic in Tehran, Iran, who received VPTs between April 2011 and October 2022. A qualified examiner (LR) collected the clinical and radiographic data for each patient/tooth from the clinic's electronic database (Dental Information System, Tarasheh Hooshmand Novin, Tehran, Iran).

### Eligibility criteria

All procedures were performed by a single endodontist (SA). The inclusion criteria were:

1. Dental pulp exposure in carious but restorable vital mature permanent teeth, with or without signs/symptoms of IP and/or AP. Traumatic pulp exposures and necrotic pulps were excluded.
2. Availability of demographic, diagnostic, procedural data, and informed consent.
3. Availability of pre-operative, immediate postoperative, and at least one follow-up radiograph (with a minimum 3-month recall) of diagnostic quality. If multiple follow-up radiographs were present, the most recent one was used for analyses.
4. Teeth that were periodontally healthy at the time of treatment, i.e., probing depths of less than 4 mm.

Records not meeting these criteria were excluded.

### Treatment protocols

The detailed treatment approaches were as follows:

#### Full pulpotomy

The pulpal vitality test (cold test with Endo-Frost; Coltène-Whaledent, Langenau, Germany) was administered before treatment, and all teeth had to show a positive response. Following local anesthesia with 2% lidocaine with 1:80,000 epinephrine (DarouPakhsh, Tehran, Iran) and isolation with cotton roll, caries was removed, exposing the pulp. If the pulp exposure exceeded 1 mm, the procedure would be a FP; if it was less than 1 mm, the procedure would be a MP. The pulp chamber roof was then excised and the coronal pulp tissue was gently eliminated with a round-end sterile diamond bur (Diatech; Coltene Whaledent, Altstatten, Switzerland), using copious irrigation with normal saline (Darou pakhsh, Tehran, Iran).

A sterile cotton pellet soaked in 5.25% sodium hypochlorite (Paxan, Tehran, Iran) was gently placed in the pulp chamber over the orifice(s) for ~ 2 min to achieve hemostasis and disinfection, and the cavity was then rinsed. The clinician proceeded with the procedure, i.e. tampon approach, even if hemostasis was not completely achieved. The pulp covering agent, calcium-enriched mixture (CEM) cement (BioniqueDent, Tehran, Iran) was then prepared according to the manufacturer's instructions and inserted into the pulp chamber by a plastic instrument with a thickness of ~ 2–3 mm, ensuring a meticulous fit and adaptation. The remaining cavity was then restored with either amalgam (Sinaloy, Teharn, Iran) or a resin-bonded composite (3M ESPE, Seefeld, Germany).

#### Miniature pulpotomy

This was the preferred treatment when dental pulp tissue exposure was ~ 1 mm^17,18^. All Procedures largely mirrored those of the FP, but only a ~ 1-mm-deep cavity was prepared into the pulp space.

#### Direct pulp capping

Procedures were similar to the FP, but after dental pulpal exposure of less than 1 mm, the site was directly covered with CEM cement.

The abovementioned VPT techniques determined as the exposure for the cohort study.

### Radiographic technique/assessment

Using paralleling techniques, digital periapical radiographic images were obtained with the same X-ray unit (Soredex, Tuusula, Finland) at 60 kVp and 7.5 mA. Beam guiding device (XCP; Kit Rinn FPS 3000, Dentsply Sirona, India), phosphor plates (PSP)/intraoral imaging plate system (Digora Optime; Soredex, Tuusula, Finland), and standardized exposure to obtain optimal and standardized diagnostic quality images. Radiographs were viewed using Windows Photo Viewer software (Microsoft, Redmont, WA, USA) on an HP Envy curved 34-inch display with a screen pixel resolution of 3440 × 1440 pixels (HP, Palo Alto, CA, USA) in a dimly lit room.

The examiner (LR) was proficient in detecting apical periodontitis and assessing PAI scores^19^ during follow-up evaluations. Consistency among the examiner was validated using Cohen’s kappa test, analyzing 50 standard radiographs.

### Data collection

The following variables were recorded in a Microsoft Excel database (Excel 2016; Microsoft, Redmond, WA, USA):

- Patient-related factors: age, and sex.
- Tooth-related factors: tooth type, and missing surfaces.
- Treatment-related factors: pulpal diagnosis, radiographic signs of AP at the time of treatment, date of treatment and date of follow-up(s), VPT type, time to achieve hemostasis, restoration type, and restoration surfaces.
- Outcome-related factors: success and failure, and date of failure/re-intervention (root canal treatment or extraction).

#### Assessment of treatment outcomes

*Survival* was defined as the tooth being present/functional (i.e. asymptomatic) at the time of follow-up. Therefore, the target event was the missing of the treated teeth due to tooth extraction. The time of survival is defined as the time between performing VPTs and the last recall if the tooth was present functional/asymptomatic in the oral cavity, OR the time of tooth extraction.

Evaluation of the treatment outcomes of VPTs was based on combined clinical and radiographic examinations. The outcome was recorded as a *success* in cases in which both the clinical/radiographic presentations were normal [i.e. Orstavik’s periapical index^19^ score of one (PAI 1)]. If patients presented either with clinical signs/symptoms (persistent pain after VPT/pain on percussion or palpation/swelling/sinus tract/non-restorable tooth fractures) or emerging PDL widening (PAI 2 and 3)/apical lesions (PAI 4 and 5)/root resorptions or persisted radiolucency without change, the outcome was recorded as a *failure*^20^. When the treatment outcome was assessed based on success and failure, the time of survival was defined as the period between the date of VPT and the date of radiographic/clinical failure. The start of the observation period was determined by the date of VPTs. If the failure occurred, the end of the observation period was defined as the date of failure or the date of the first recall/radiograph in which the evidence of failure was found.

### Sample size calculation

For multivariate regression analyses in clinical research, an event-per-variable ratio of over 10 is recommended to ensure accuracy and statistical power^21^. Considering our evaluation of 9 variables, we estimated a need for over 90 teeth for optimal analysis.

### Statistical analysis

We employed descriptive statistics to characterize study cases. Patient, tooth, and treatment factors were statistically analyzed concerning the dichotomous outcome of success versus failure using a Cox proportional hazards model. Mean survival times were determined through Kaplan–Meier curves/analysis and the Log-rank test was utilize to compare survival across the three treatment groups. Sensitivity and model fitness were assessed using the Omnibus test of model and the − 2 log likelihood ratio (− 2LL), a metric in maximum likelihood estimation for comparing different models and determining the best fit for a given dataset. The statistical analyses were conducted using IBM SPSS Statistics for Windows Version 21.0 (IBM, Armonk, NY, USA), with significance set at *P* < 0.05.

### Ethics approval and consent to participate

Ethical approval was obtained from the Ethics Committee of the Research Institute for Dental Sciences of Shahid Beheshti University of Medical Sciences (IR.SBMU.DRC.REC.1401.116). Informed consent was obtained from the patients before the treatments.

## Results

**Figure 1:**
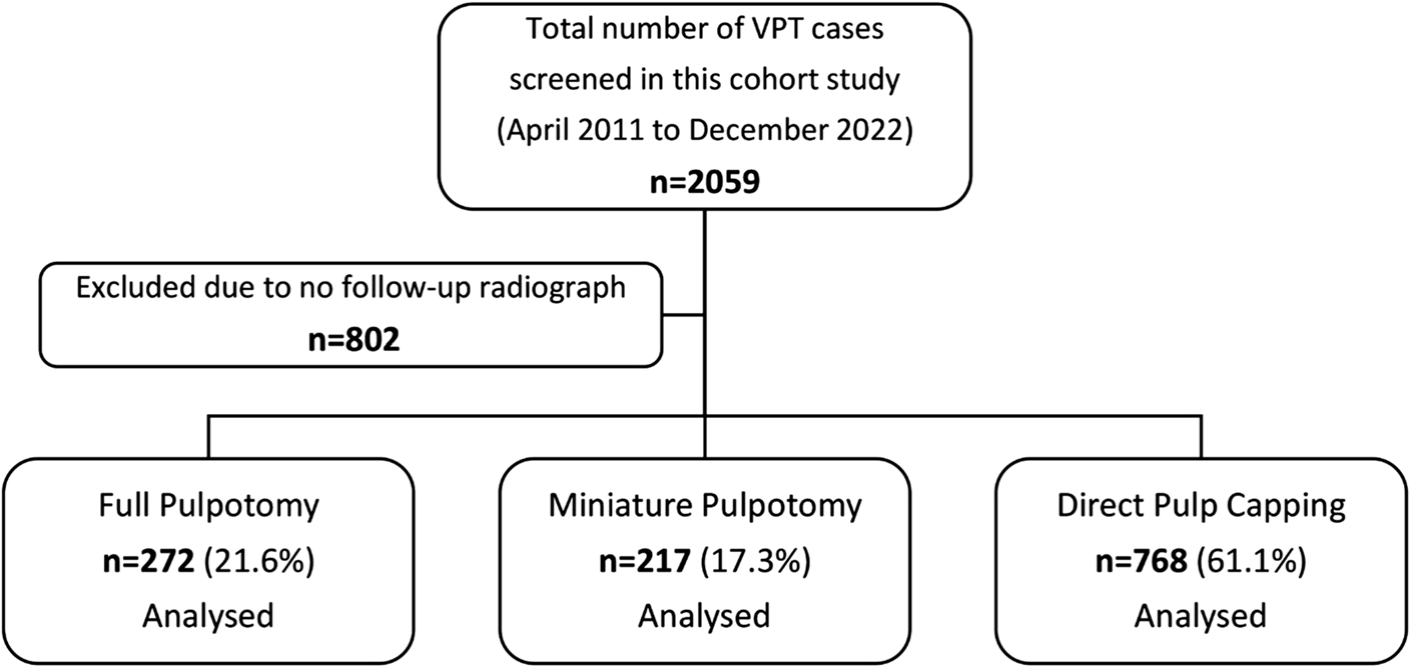
A diagrammatic flowchart illustrating the cases studied inVPT.

From April 2011 to December 2022, 2059 VPT-treated teeth in 1567 patients were included. Of these, 1257 VPTs from 1149 patients met the inclusion criteria and were incorporated into the analyses (Fig. 1). There were no missing data, and all included teeth were analyzed using a complete case approach.

**Figure 2:**
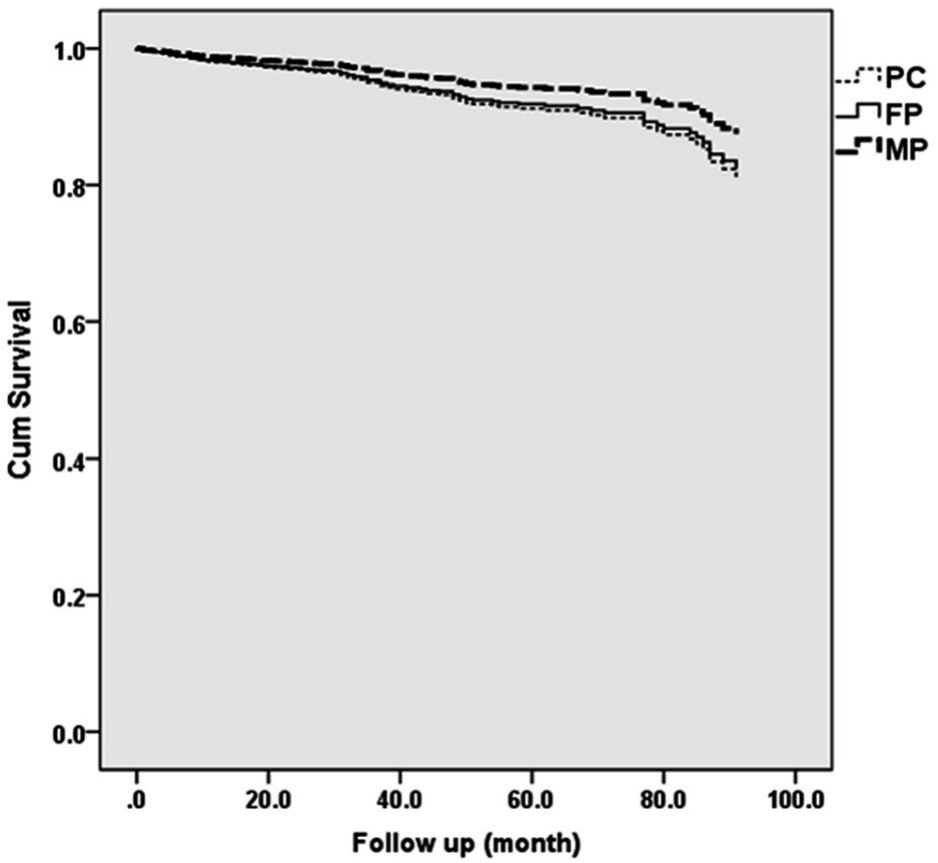
Kaplan–Meier survival curves depicting tooth survival of three VPTs (*PC* direct pulp capping, *MP* miniature pulpotomy, *FP* full pulpotomy).

The patient cohort consisted of 65.79% females (mean ± SD age: 41.27 ± 11.63 years) and 34.21% males (mean ± SD age: 41.59 ± 10.74 years). The average follow-up duration was 42.21 months (median: 39.00 months), with an overall 116-month survival rate of 99.1% and success rate of 91.6%. The DPC, MP, and FP groups had a mean (± SE) estimated survival times (in months) of 102.27 ± 1.46 (95% CI 99.40–105.14), 104.26 ± 2.66 (95% CI 99.02–109.49), and 99.43 ± 2.99 (95% CI 93.57–105.30) respectively. No significant differences were observed amongst these groups (*P* = 0.363). The Kaplan–Meier survival curves for the outcomes of DPC, MP, and FP are depicted in Fig. 2.

**Figure 3:**
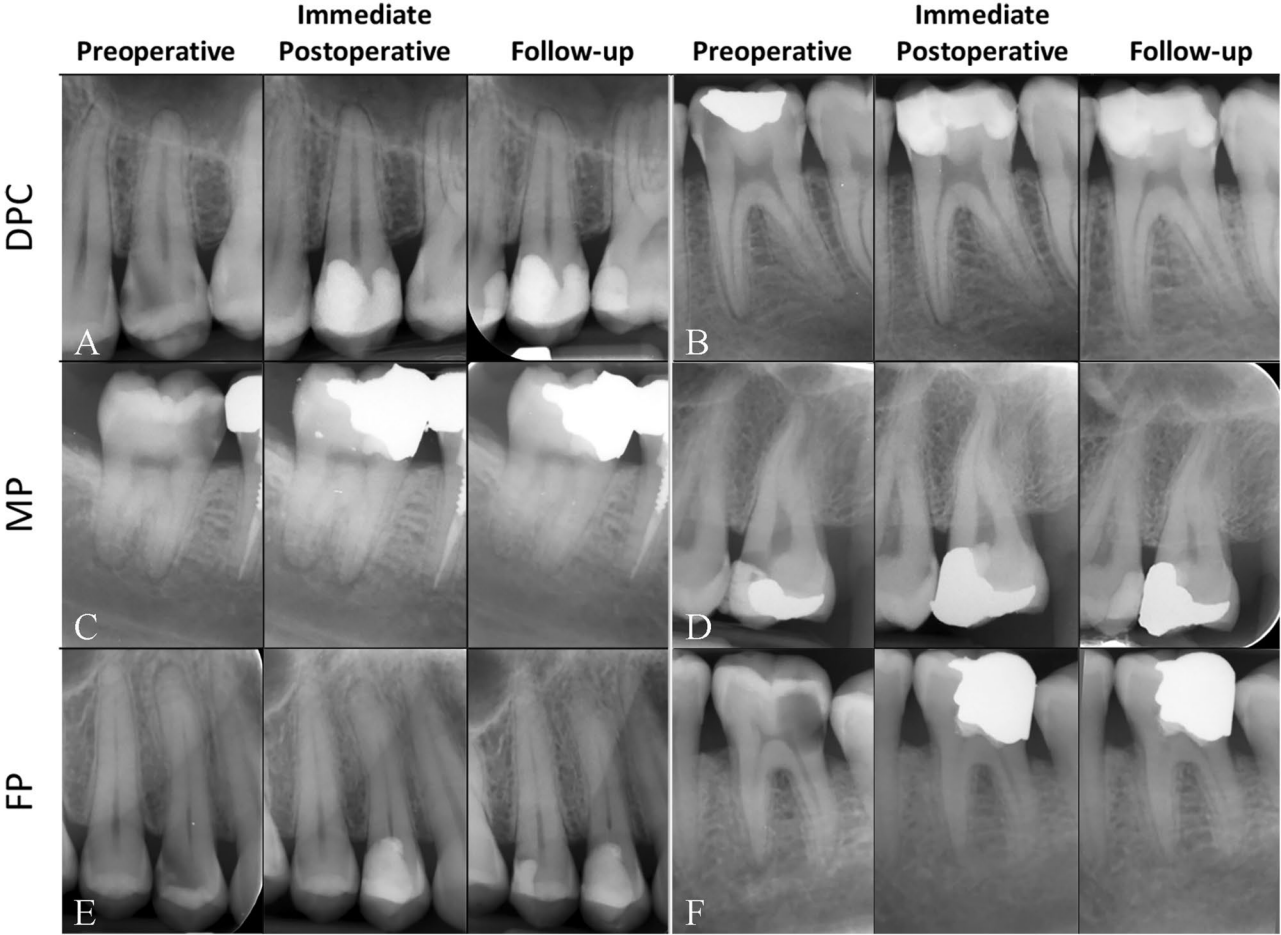
Examples of successful cases with different types of VPT: (**A**) DPC of an upper second premolar in a 32-year-old male with a 54-month follow-up; (**B**) DPC of a lower first molar in a 25-year-old male with a 26-month follow-up; (**C**) MP of a lower second molar in a 37-year-old female with a 33-month follow-up; (**D**) MP of an upper third molar in a 35-year-old male with a 45-month follow-up; (**E**) FP of an upper first premolar in a 38-year-old female with a 46-month follow-up; (**F**) FP of a lower first molar in a 35-year-old female with a 31-month follow-up.

**Figure 4:**
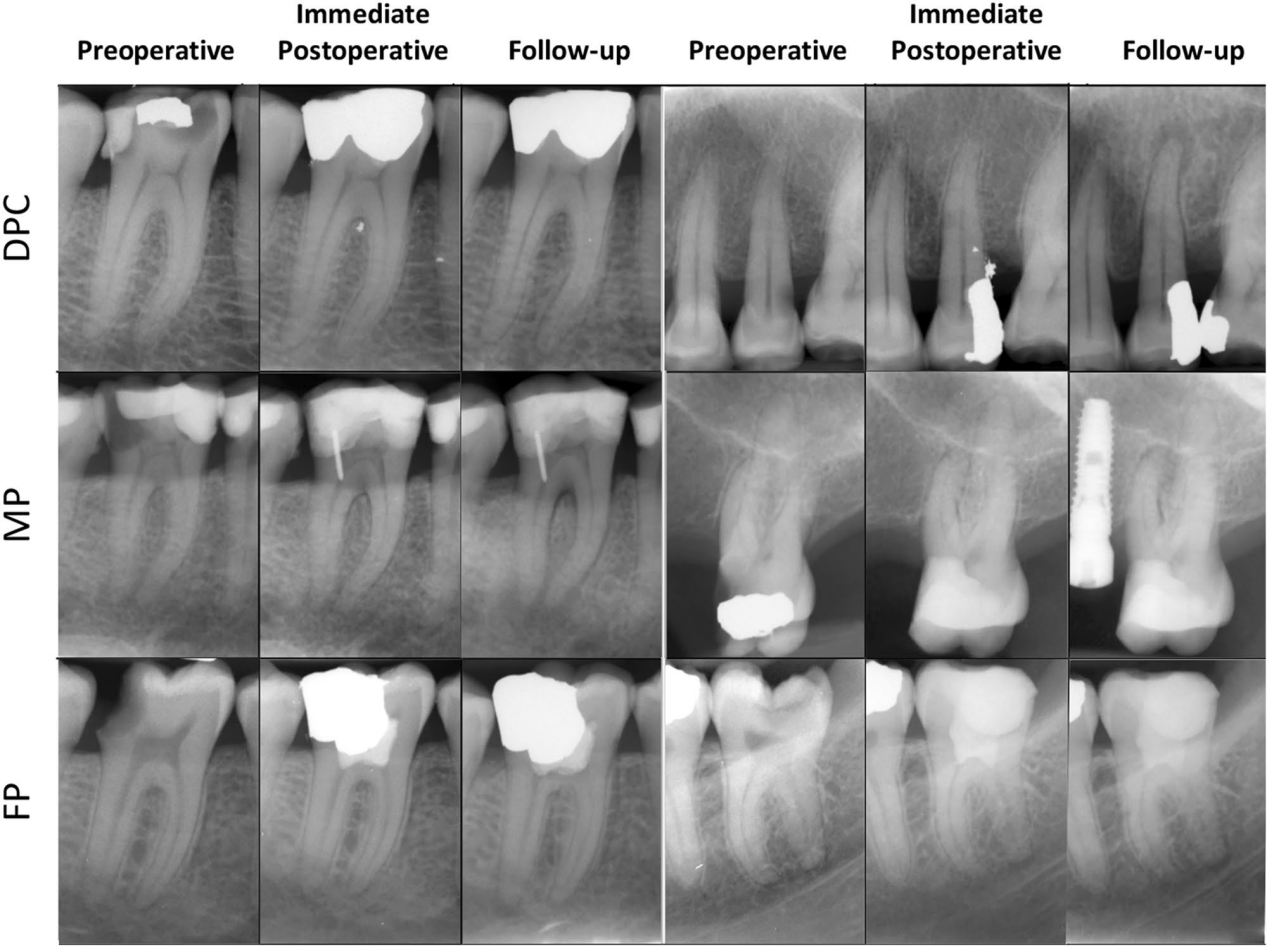
Examples of failure cases with different types of VPT: (**A**) DPC of a lower first molar in a 50-year-old male with a 34-month follow-up; (**B**) DPC of an upper second premolar in a 61-year-old female with a 87-month follow-up; (**C**) MP of a lower first molar in a 54-year-old female with a 10-month follow-up; (**D**) MP of an upper first molar in a 54-year-old female with an 8-month follow-up; (**E**) FP of a lower first molar in a 26-year-old male with a 39-month follow-up; (**F**) FP of a lower third molar in a 29-year-old female with a 48-month follow-up.

**Figure 5:**
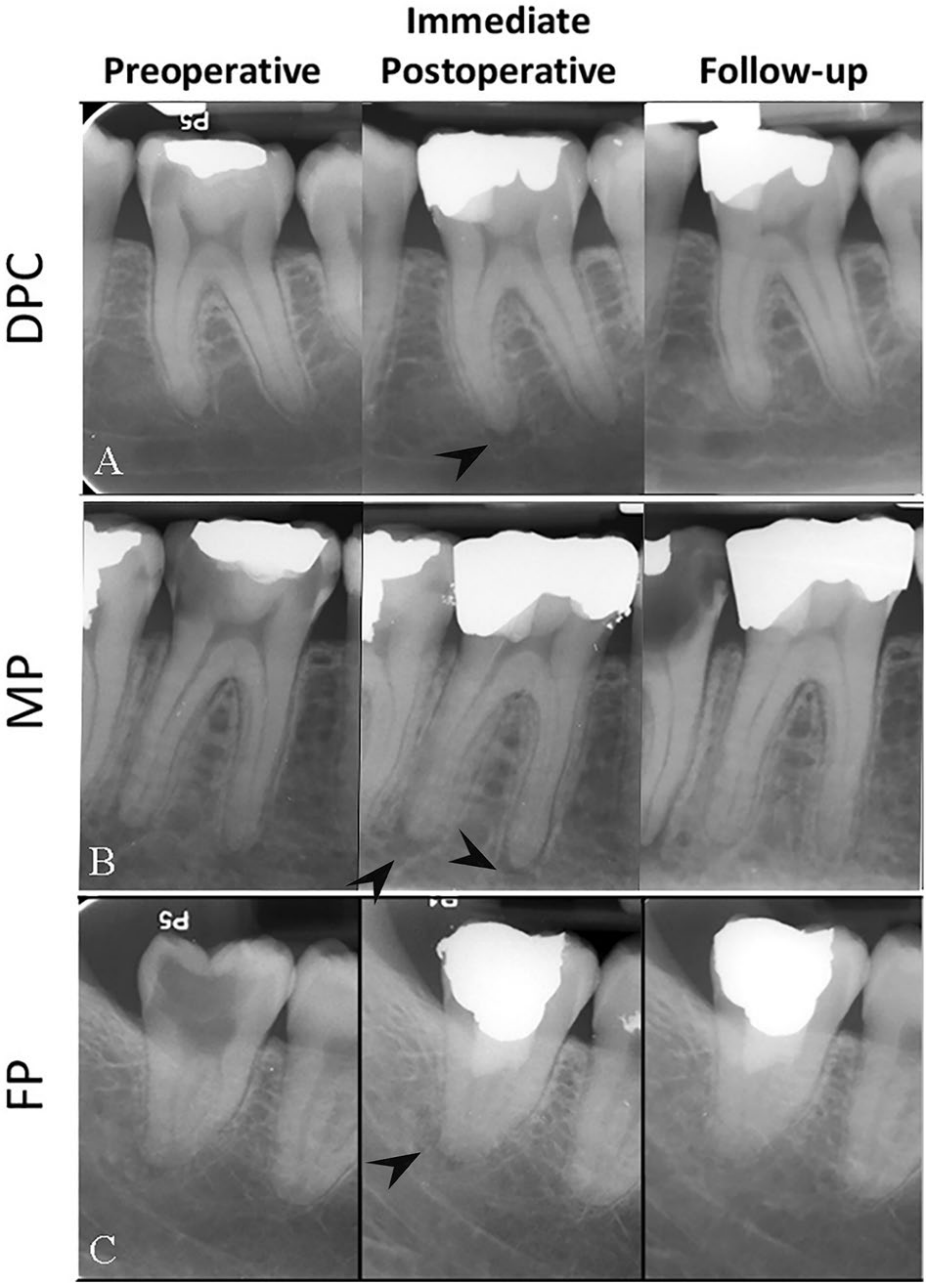
Examples of the presence of apical periodontitis (PAI-3; black arrow heads) before VPTs with successful clinical outcomes: (**A**) DPC of a lower first molar in a 57-year-old female with a 59-month follow-up; (**B**) MP of a lower first molar in a 35-year-old male with a 61-month follow-up; (**C**) FP of a lower third molar in a 39-year-old female with an 18-month follow-up.

Factors related to patients, teeth, and treatment in the three study groups are detailed in Table 1. The intra-observer agreement for the examiner (LR) was notably strong (kappa = 0.847). Representative images of both successful and unsuccessful treatments are displayed in Figs. 3 and 4, respectively. Additionally, treated cases with concurrent IP and AP, totaling 135 in number and diagnosed with a PAI of 3, demonstrated an overall success rate of 78.5% (Table 1 and Fig. 5).

**Table 1:** Patient-, tooth- and treatment-related factors in the three study groups categorized by outcomes.

Study Groups	FP		MP		DPC		Total	
Factors	Outcomes							
	Success	Failure	Success	Failure	Success	Failure	Success	Failure
Age (Mean Years ± SD)	41.63 ± 11.21	43.63 ± 11.26	41.07 ± 11.12	44.94 ± 11.18	41.12 ± 11.37	42.37 ± 12.20	41.22 ± 11.27	43.09 ± 11.74
Gender, N (%)								
Male	61 (85.9)	10(14.1)	81 (95.3)	4 (4.7)	250 (91.2)	24 (8.8)	392 (91.2)	38 (8.8)
Female	184 (91.5)	17 (8.5)	120 (90.9)	12 (9.1)	456 (92.3)	62 (7.7)	760 (91.9)	67 (8.1)
Tooth Type, N (%)								
Molars								
Upper	100 (96.2)	4 (3.8)	80 (92.0)	7 (8.0)	218 (95.2)	11 (4.8)	398 (94.8)	22 (5.2)
Lower	49 (87.5)	7 (12.5)	94 (94.0)	6 (6.0)	258 (90.8)	26 (9.2)	401 (91.1)	39 (8.9)
Premolars								
Upper	72 (88.9)	9 (11.1)	16 (88.9)	2 (11.1)	156 (92.9)	12 (7.1)	244 (91.4)	23 (8.6)
Lower	20 (76.9)	6 (23.1)	10 (90.9)	1 (9.1)	64 (84.2)	12(15.8)	94 (83.2)	19(16.8)
Incisors								
Upper/Lower	4 (80.0)	1 (20.0)	1 (100.0)	0 (0.0)	10 (90.9)	1 (9.1)	15 (88.2)	2 (11.8)
No Hemostasis (in ~ 2 min), N (%)	159 (90.3)	17 (9.7)	150 (93.2)	11 (6.8)	77 (89.5)	9 (10.5)	386 (91.3)	37 (8.7)
Presence of Preoperative Restoration, N (%)	124 (91.2)	12 (8.8)	119 (90.8)	12 (9.2)	392 (93.1)	29 (6.9)	635 (92.3)	53 (7.7)
Diagnosis, N (%)								
Symptomatic Irreversible Pulpitis (SIP)	204 (89.9)	23 (10.1)	169 (92.9)	13 (7.1)	273 (87.2)	40 (12.8)	646 (89.5)	76 (10.5)
Radiographic sign of Apical Periodontitis (AP) (PAI = 2 or 3)	132 (87.4)	19 (12.6)	105 (88.2)	14 (11.8)	222 (85.4)	38 (14.6)	459 (86.6)	71 (13.4)
Cases with Concurrent SIP and AP (PAI = 2)	79 (89.8)	9 (10.2)	51 (87.9)	7 (12.1)	70 (86.4)	11 (13.6)	200 (88.1)	27 (11.9)
Cases with Concurrent SIP and AP (PAI = 3)	30 (81.1)	7 (18.9)	41 (89.1)	5 (10.9)	35 (67.3)	17 (32.7)	106 (78.5)	29 (21.5)
Restorative Material, N (%)								
Amalgam	120 (94.5)	7 (5.5)	109 (95.6)	5 (4.4)	319 (93.3)	23 (6.7)	548 (94.0)	35 (6.0)
Composite Resin	125 (86.2)	20 (13.8)	92 (89.3)	11 (10.7)	387 (90.8)	39 (9.2)	604 (89.6)	70 (10.4)
Restorative Surfaces, N (%)								
One	1 (50.0)	1 (50.0)	3 (100.0)	0 (0.0)	32 (94.1)	2 (5.9)	36 (92.3)	3 (7.7)
Two	126 (90.6)	13 (9.4)	127 (95.5)	6 (4.5)	443 (92.9)	34 (7.1)	696 (92.9)	53 (7.1)
Three	101 (89.4)	12(10.6)	62 (87.3)	9 (12.7)	218 (91.2)	21 (8.8)	381 (90.1)	42 (9.9)
Four	17 (94.4)	1(5.6)	9 (90.0)	1 (10.0)	13 (72.2)	5 (27.8 )	39 (84.8)	7 (15.2)
Follow-up Time, (Mean Months ± SD)	41.41 ± 25.63	32.15 ± 23.75	44.17 ± 25.77	31.06 ± 24.79	43.23 ± 27.17	34.58 ± 28.41	43.01 ± 26.60	33.42 ± 26.56
Outcomes, N (%)	245 (90.1)	27 (9.9)	201 (92.6)	16 (7.4)	706 (91.9)	62 (8.1)	1152 (91.6)	105 (8.4)
Survival, N (%)	267 (98.2)	5 (1.8)	214 (98.6)	3 (1.4)	765 (99.6)	3 (0.4)	1246 (99.1)	11 (0.9)

Cox proportional hazards model analyses revealed significant correlations between symptomatic IP (HR = 1.974, 95% CI 1.242–3.137; *P* = 0.004), radiographic signs of AP (HR = 2.983, 95% CI 1.961–4.540; *P* < 0.000), restoration type (HR = 2.263, 95% CI 1.423–3.600; *P* = 0.001), and restoration surfaces (HR = 1.401, 95% CI 1.034–1.899; *P* = 0.030) with the outcomes of VPTs. Other factors were not statistically significant (Table 2).

**Table 2:** Results of Cox regression analyses for success in vital pulp therapies.

	Beta	SE	Wald	df	*P*-value	Adjusted Hazard Ratio	95% CI	
							Lower	Upper
Incisors*^1^			8.898	4	0.064			
Lower Molars vs. Incisors	0.417	0.751	0.308	1	0.579	1.517	0.348	6.606
Lower Premolars vs. Incisors	0.867	0.749	1.341	1	0.247	2.379	0.549	10.320
Upper Molars vs. Incisors	-0.018	0.756	0.001	1	0.981	.982	0.223	4.324
Upper Premolars vs. Incisors	0.138	0.743	0.034	1	0.853	1.148	0.268	4.923
Previous Restoration+	0.208	0.208	1.007	1	0.316	1.232	0.820	1.851
Symptomatic IP vs. Reversible Pulpitis	0.680	0.236	8.270	1	0.004	1.974	1.242	3.137
AP vs. Normal PDL	1.093	0.214	26.047	1	0.000	2.983	1.961	4.540
Hemostasis+	0.069	0.287	0.057	1	0.811	1.071	0.611	1.878
MP*^2^			1.725	2	0.422			
DPC vs. MP	0.453	0.347	1.708	1	0.191	1.573	0.797	3.105
FP vs. MP	0.286	0.331	0.744	1	0.388	1.331	0.695	2.546
Composite Resin vs. Amalgam	0.817	0.237	11.900	1	0.001	2.263	1.423	3.600
Filling Surfaces	0.337	0.155	4.733	1	0.030	1.401	1.034	1.899

The effect of age, and sex, which could be confounding demographic factors, were adjusted in the Cox proportional hazards model. (+ Being positive for or having that event; *1 Reference category: Incisors; *2 Reference category: Miniature Pulpotomy).

Interactions between independent variables such as “age and periapical Dx”, “periapical Dx and pulp Dx”, “hemostasis and VPT type”, and “filling surface and restoration type” were explored. The only independent variables showing interaction were VPT type and the number of restoration surfaces. Specifically, for DPC cases, an increase in the number of restoration surfaces significantly impacted treatment failures (*P* = 0.023, HR = 1.578). When comparing DPC to FP and MP, the P-values were 0.652 and 0.163, respectively.

The validity of the model used in this investigation was assessed using the omnibus Chi-square test, which demonstrated significant model fitness (*X*_2_ = 76.189, *P* < 0.001). Additionally, the log-likelihood ratio was utilized to further evaluate the model's fit, yielding a value of − 2LL = 1268.232.

## Discussion

In the context of our null hypothesis that explored the impact of various factors on the outcomes of VPTs, this study presents the first large-scale (> 1250 teeth), long-term retrospective cohort investigation into VPTs. Our comprehensive examination of factors affecting outcomes revealed that symptomatic IP, radiographic signs of AP at treatment onset, increased missing surfaces, and composite restorations emerge as negative predictors of VPT outcomes. Despite inherent limitations, these findings contribute valuable insights into the intricate dynamics influencing the efficacy of VPTs.

Due to the difficult and expensive nature, low patient attendance and time-consuming processes of long-term randomized clinical trials, the majority of investigations in the field of VPT have enrolled a maximum of 146 patients and set ≤ 2-year intervals to examine the final success rates^22–24^. Such short to intermediate-term randomized clinical trials may inadvertently inflate success rates, potentially overlooking instances of regression. In one study, a notable 14.7% decline in radiographic success rates was observed between the years first and fifth^10,25,26^. Retrospective studies on VPT, due to their more streamlined nature, tend to present larger sample sizes. However, previous highs reported are 205 and 273 cases^9,27^. Our study overshadows these, detailing the outcomes of 1,257 cases over 10 years, setting a new benchmark for retrospective studies of this nature.

The success rate for VPTs in our study was 91.6%, agreeing with a recent meta-analysis on VPT of teeth affected by both reversible and irreversible pulpitis^6^. Furthermore, the outcomes of the three distinct VPT types showed no statistically significant differences, a finding that echoes prior research in this domain^18^.

Earlier studies have documented a 97% survival rate for 1,462,936 endodontically treated teeth over a period of 8 years^28^. This rate is the highest amongst similar studies with smaller sample size which reported up to ~ 90% survival rates for these teeth^29^. Yet, the long-term outcomes post-VPT remained uncharted until now. Our research provides clarity, showcasing an impressive 99.1% survival rate over a mean of ~ 42 months. Importantly, there were no significant differences observed between the survival rates of teeth undergoing FP, MP, and DPC.

The age of patients, which spanned from 12 to 77 years, did not emerge as a prognostic factor for VPT outcomes. This observation is consistent with prior clinical trials and systematic reviews, underscoring the efficacy of VPTs across all age groups, including the elderly^23,30,31^. Additionally, gender did not influence VPT outcomes, a conclusion in harmony with earlier studies^9,32^.

Interestingly, our findings indicate that the type of tooth does not significantly influence VPT outcomes. While numerous previous studies have predominantly focused on a single tooth type, often emphasizing molars^10,24,27,30,33^, only a one study has considered more than two types^34^. However, the study did not thoroughly investigate tooth type as a factor affecting prognosis.

Calcium-silicate-based endodontic biomaterials such as mineral trioxide aggregate (MTA), Biodentine, and CEM cement have been recommended as the first choices for pulpotomy in permanent mature teeth^35^. In this study, we employed CEM cement with similar clinical applications to MTA but with a different chemical composition. It offers benefits such as a shorter setting time^36^ and improved antibacterial properties^37^. There is no clinical report to show the tooth discoloration effect of this cement. In terms of the clinical applications of CEM cement for VPTs, there are many reported studies that confirm the successful results in IPC, DPC, MP, PP, and FP of permanent teeth^4,11,18^ as well as primary molars^38^.

We explored the extent of caries, focusing on missing walls or filling surfaces, as a potential prognostic factor. A connection was identified, though the presence of preoperative restorations did not affect the outcomes. Contrary to our findings, two previous trials in primary teeth found that the extent of missing/filled surfaces did not impact the success rate of VPT^39,40^.

While we deviate from conventional practice^41^, by not employing rubber dam isolation in our VPT procedures, our alternative approach, integrating cotton roll isolation, high-vacuum suction, and cavity disinfection with NaOCl, yielded promising results. While it is critical to acknowledge that the universal acceptance of rubber dam usage may not be practical in resource-constrained areas, where expensive dental instruments are often inaccessible, it is important to recognize the benefits of using it in preventing contamination. Our study aligns with a broader global goal of offering practical solutions in such contexts. Duncan et al. have also rightly emphasized the need for deeper exploration into the impact of rubber dam isolation on VPT outcomes^42^. Our pragmatic and accessible approach contributes to ongoing discussions about making dental interventions, like VPT, more feasible and applicable in diverse global settings. This discussion invites consideration of effective alternatives in situations where conventional practices face limitations.

The existing literature on bleeding time’s impact on VPT outcomes offers mixed insights. While some studies postulate a link between bleeding time and the condition of pulpal tissue^20,43^, others, such as a systematic review, contend that there is no established correlation between the two^7,35^. Our findings lean towards the latter, suggesting that achieving hemostasis is not a pivotal factor for VPT outcomes^33,44^. The results of the current study also showed that the tampon approach^45^ may be employed with successful outcomes, similar to previous reports^44,46^.

While current evidence underscores the critical role of coronal restoration in ensuring the long-term success of VPTs^47^, our study substantiates and extends this finding. The choice between amalgam and composite resin restorations emerged as a significant factor influencing success rates, favoring amalgam restorations over composite resin restorations^9^. In elucidating this preference, we carefully weighed various patient-centered factors, including patient preferences, the extent of caries, potential aesthetic concerns, and overall oral health status. These considerations were pivotal in our decision-making process, emphasizing the importance of tailoring dental interventions to individual patient needs.

Traditionally, the infected non-vital pulp has been deemed the primary cause of radiographic signs of AP. These signs have conventionally led to an inclination for the immediate initiation of RCT, particularly when an IP diagnosis is considered. However, recent research challenges this perspective, suggesting that even symptomatic IP associated with AP can be successfully treated with various VPT techniques^26^. Supporting this perspective, a recent review highlights successful VPT outcomes in vital teeth with associated AP, with various studies reporting positive results, including periapical healing and hard tissue bridge formation^42^. These findings collectively underscore a paradigm shift in understanding the relationship between AP and vital teeth diagnosed with IP, indicating that such involved teeth can be treated successfully using various VPT techniques In our study, ~ 42% of cases treated with VPTs exhibited radiographic evidence of AP, with PAI values of 2 (n = 227) and 3 (n = 135). Notably, the latter displayed a 78% success rate, representing a 14% decrease from the overall success rate reported in our study. Furthermore, cases manifesting these signs at the onset of treatment were linked to increased VPT failure rates over time. Specifically, VPT in cases with AP (PAI = 2 or 3) exhibited ~ 3 times the failure rate compared to those with a normal periapical status. While these findings align with other research, it is essential to acknowledge potential variations in rates. Importantly, we adhered to rigorous criteria for radiographic success (PAI 1), and any case with PAI ≥ 2 was deemed a failure. These findings contribute to our understanding of the success rates of VPTs in cases with radiographic evidence of AP.

It is crucial to understand the inherent biases present in retrospective cohort studies, such as those related to selection, performance, detection, and attrition. The authenticity of such studies largely relies on comprehensive patient records. For this study, only teeth possessing complete radiographic and clinical records (n = 1257) were chosen for analysis. Consequently, we effectively mitigated the risks of selection and attrition biases. Moreover, the expansive cohort size, approximately 14 times the estimated sample size, further reduced potential selection bias. However, it is important to highlight that in our study, neither the patients, the operator, nor the examiner were blinded. This non-blinding introduces the common retrospective study challenges of performance and detection bias. Notably, all VPT procedures, including DPC, MP, and FP, were consistently conducted by a singular endodontist (SA), thereby minimizing operator variability and potential bias.

Last but not least, in our examination of endodontic treatment efficacy, two key studies stand out. Kielbassa et al.’s observational study on a lower Austrian subpopulation (22,586 teeth) revealed a significant correlation between suboptimal RCT and periapical pathosis^12^. Their findings stressed the importance of comprehensive endodontic and advanced restorative approaches, highlighting the positive impact of posts/screws on periapical health. Simultaneously, our cross-sectional survey in an Iranian population found 52% of endodontically treated teeth presenting with AP, emphasizing the link between poor RCT/coronal filling and increased AP incidence^13^. Importantly, the real-world quality of complex RCT often falls short with a high failure rate when performed by general dentists. In contrast, our study strongly recommends the more universal adoption of the remarkably simple and effective VPT in routine dental practice to address these challenges.

## Conclusions

This comprehensive retrospective cohort study, spanning over a decade and encompassing > 1250 treated teeth with diverse tooth types, offers robust evidence on the long-term success and survival rates of various VPTs (DPC, MP, FP) in managing carious pulp exposures with/without signs/symptoms of IP and/or AP. The observed > 99% survival rate and ~ 92% success rate emphasize the efficacy of VPTs, even in the presence of symptomatic IP, radiographic signs of AP at treatment onset, caries extent, and the use of composite restorations as negative outcome predictors. Notably, our study highlights the practicality of various simple VPTs by demonstrating that they can be performed successfully without employing a rubber dam, particularly in resource-limited settings. Additionally, in continuous bleeding cases, the tampon approach yields outcomes comparable to bleeding control cases treated with the standard approach. Moreover, these findings provide valuable insights for clinicians with practical guidance for adopting various VPTs across a diverse range of patient demographics and clinical scenarios. This evidence supports practitioners’ confidence in incorporating VPTs into their routine treatment strategies worldwide.

While the findings from this study serve as a foundation for advancing our understanding of VPT outcomes and refining treatment approaches for optimal patient care, future research endeavors may delve into nuanced factors influencing VPT success/survival.

## Data Availability

The data that support the findings of the current study are available from the corresponding author upon reasonable request.
